# Supplementary material for: A comprehensive approach to predicting weight gain and therapy response in psychopharmacologically treated major depressed patients: A cohort study protocol
Source: PLoS One. 2022 Jul 21;17(7):e0271793. doi: 10.1371/journal.pone.0271793 (PMC9302848; doi:10.1371/journal.pone.0271793)
Supplement: Attachment — Submitted filename: Response to Reviewers.docx [file pone.0271793.s001.docx]

Maria S Simon

Department of Psychiatry and Psychotherapy,

University Hospital, Ludwig-Maximilians-University,

80336 Munich, Germany

Maria.Simon@med.uni-muenchen.de

Editor-in-Chief Dr. E. Chenette

Staff Editor H. Landenmark

PLOS ONE

12 Jun 2022

Dear Editors,

Thank you very much for giving our group the opportunity to submit a revised version of our manuscript. We have addressed the editorial comments and taken the reviewers’ comments into serious consideration. Please find our answers to the editorial and reviewer comments listed below. The changes to the manuscript are marked in yellow inside the revised manuscript document as well. Further, the figure was uploaded to PACE and submitted as appropriate.

The amended competing interest statement now reads as follows: I have read the journal's policy and the authors of this manuscript have the following competing interests: RM has received reimbursement by Janssen-Cilag, Emalex Biosciences, Boehringer-Ingelheim, and Oryzon for carrying out studies, and has received speaker’s honoraria from Otsuka over the past five years, all outside the submitted work. No other potential or aactual financial or non-financial competing interests apply. MSS, BB, and CG have declared that no competing interests exist. This does not alter our adherence to PLOS ONE policies on sharing data and materials.

Regarding data availability, no data has been produced so far since the present manuscript is a study protocol. The statement was adjusted as follows: A minimal anonymized data set will be uploaded to a publicly accessible repository upon publication of data evaluations produced by this project.

All authors approved the final version of the manuscript and agreed to publication. I hope you find this manuscript now suitable for publication in your journal.

Yours sincerely and on behalf of the other authors,

Maria S. Simon

Editorial comments:

1. Please ensure that your manuscript meets PLOS ONE's style requirements, including those for file naming.

Style was checked again and is in compliance with the requirements.

2. We note that the grant information you provided in the ‘Funding Information’ and ‘Financial Disclosure’ sections do not match. When you resubmit, please ensure that you provide the correct grant numbers for the awards you received for your study in the ‘Funding Information’ section.

As is appears from the author guidelines, the funding information and the financial disclosures mean the same section. The authors received no specific funding for this work. Initially, we gave information about a planned funding application. However, no funding was installed so far by any third party. Thus, there are no grant numbers to report. So, we just leave it to the given statement.

3. Thank you for stating the following in the Competing Interests section: "I have read the journal's policy and the authors of this manuscript have the following competing interests: RM has carried out studies financed by Janssen-Cilag, Emalex, Boehringer-Ingelheim and has received speaker’s honoraria from Otsuka over the past three years. MSS, BB, and CG have declared that no competing interests exist." We note that you received funding from a commercial sources: Janssen-Cilag, Emalex, Boehringer-Ingelheim and Otsuka.

Please provide an amended Competing Interests Statement that explicitly states this commercial funder, along with any other relevant declarations relating to employment, consultancy, patents, products in development, marketed products, etc. Within this Competing Interests Statement, please confirm that this does not alter your adherence to all PLOS ONE policies on sharing data and materials by including the following statement: "This does not alter our adherence to PLOS ONE policies on sharing data and materials.”

If there are restrictions on sharing of data and/or materials, please state these. Please note that we cannot proceed with consideration of your article until this information has been declared. Please include your amended Competing Interests Statement within your cover letter. We will change the online submission form on your behalf.

We have now amended the competing interests statement as follows:

I have read the journal's policy and the authors of this manuscript have the following competing interests: RM has received reimbursement by Janssen-Cilag, Emalex Biosciences, Boehringer-Ingelheim, and Oryzon for carrying out studies, and has received speaker’s honoraria from Otsuka over the past five years, all outside the submitted work. No other potential or aactual financial or non-financial competing interests apply. MSS, BB, and CG have declared that no competing interests exist. This does not alter our adherence to PLOS ONE policies on sharing data and materials.

4. In your Data Availability statement, you have not specified where the minimal data set underlying the results described in your manuscript can be found. PLOS defines a study's minimal data set as the underlying data used to reach the conclusions drawn in the manuscript and any additional data required to replicate the reported study findings in their entirety. All PLOS journals require that the minimal data set be made fully available. For more information about our data policy, please see <http://journals.plos.org/plosone/s/data-availability>. Upon re-submitting your revised manuscript, please upload your study’s minimal underlying data set as either Supporting Information files or to a stable, public repository and include the relevant URLs, DOIs, or accession numbers within your revised cover letter. For a list of acceptable repositories, please see http://journals.plos.org/plosone/s/data-availability#loc-recommended-repositories. Any potentially identifying patient information must be fully anonymized. Important: If there are ethical or legal restrictions to sharing your data publicly, please explain these restrictions in detail. Please see our guidelines for more information on what we consider unacceptable restrictions to publicly sharing data: <http://journals.plos.org/plosone/s/data-availability#loc-unacceptable-data-access-restrictions>

Note that it is not acceptable for the authors to be the sole named individuals responsible for ensuring data access. We will update your Data Availability statement to reflect the information you provide in your cover letter.

Since the present manuscript is a study protocol, no data was generated and evaluated in this manuscript. Thus, there is no data that can be made available. However, the authors state that upon later publication of data evaluations from this project, the minimal data set will be uploaded to a publicly accessible repository. The statement was changed in the text.

5. We note that you have indicated that data from this study are available upon request. PLOS only allows data to be available upon request if there are legal or ethical restrictions on sharing data publicly. For more information on unacceptable data access restrictions, please see <http://journals.plos.org/plosone/s/data-availability#loc-unacceptable-data-access-restrictions>

In your revised cover letter, please address the following prompts:

a) If there are ethical or legal restrictions on sharing a de-identified data set, please explain them in detail (e.g., data contain potentially sensitive information, data are owned by a third-party organization, etc.) and who has imposed them (e.g., an ethics committee). Please also provide contact information for a data access committee, ethics committee, or other institutional body to which data requests may be sent.

b) If there are no restrictions, please upload the minimal anonymized data set necessary to replicate your study findings as either Supporting Information files or to a stable, public repository and provide us with the relevant URLs, DOIs, or accession numbers. For a list of acceptable repositories, please see <http://journals.plos.org/plosone/s/data-availability#loc-recommended-repositories>. We will update your Data Availability statement on your behalf to reflect the information you provide.

Please see the answer for comment number 4.

6. Please include captions for your Supporting Information files at the end of your manuscript, and update any in-text citations to match accordingly. Please see our Supporting Information guidelines for more information: <http://journals.plos.org/plosone/s/supporting-information>.

There are no Supporting Information files with this submission.

7. Please review your reference list to ensure that it is complete and correct. If you have cited papers that have been retracted, please include the rationale for doing so in the manuscript text, or remove these references and replace them with relevant current references. Any changes to the reference list should be mentioned in the rebuttal letter that accompanies your revised manuscript. If you need to cite a retracted article, indicate the article’s retracted status in the References list and also include a citation and full reference for the retraction notice.

The reference list was checked again. One reference was updated as it was accepted for publication in the meantime (reference number 33).

Reviewer comments:

Thank you very much for your review and pointing out unclarities. We have now revised the manuscript according to the comments by improving explanations in the appointed sections.

1. Introduction, Objectives and hypotheses: The authors indicate that they will evaluate circadian rhythms, but it seems more pertinent to indicate that they will only evaluate the chronotype (Lines 136 and 151).

Indeed, in the introduction we speak of circadian rhythm more generally as this is what can be found in the literature. Coming to the objectives, we have now clarified that chronotype as a proxy for individual circadian rhythm will be measured. This was also changed in the abstract.

2. Objectives and hypothesis: They indicate three main hypotheses, in the first of these (lines 157-159), they indicate “a depressed patient subgroup. They mean that they will evaluate the subtypes of depression? In any case, to which subgroup do they refer?

I do not find in the introduction the support of this hypothesis.

We reformulated the term “depressed patient subgroup” to make it clearer that we hypothesize to find a pattern of the mentioned variables expressed a certain way, thereby characterizing some of the investigated patients as a new subgroup. Further, an additional sentence was inserted in the objectives to better explain the line of reasoning for analyzing the mentioned variables conjointly. As for reference of the hypothesized variable expressions in association to other variables, please see the introduction.

3. Materials and Methods: Eligibility criteria. It would be useful if they indicated what are the criteria for hospitalization "psychiatric inpatients". Not all readers are familiar with psychiatric treatments, define acronyms for ECT.

This is a naturalistic observational design. Thus, patients are included in the study as they naturalistically are hospitalized. To get a better impression of this patient group, we have added some very common reasons for why depressed patients may be hospitalized. Abbreviation is now written in full and the whole text was checked again for unexplained acronyms.

4. Outcomes: The authors only indicate two outcomes: “body weight” and “depression symptom change”, but it is not clear what response they will evaluate with respect to the first hypothesis (lines 157-159) when referring to “a depressed patient subgroup”.

The outcomes for the first hypothesis are all the named variables since analyses will be of correlative nature. We have added an explanation to the outcomes and better clarified primary and secondary outcomes.
